# Cost-effectiveness analysis of myopia management: A systematic review

**DOI:** 10.3389/fpubh.2023.1093836

**Published:** 2023-02-27

**Authors:** Sylvia Agyekum, Poemen P. Chan, Yuzhou Zhang, Zhaohua Huo, Benjamin H. K. Yip, Patrick Ip, Clement C. Tham, Li Jia Chen, Xiu Juan Zhang, Chi Pui Pang, Jason C. Yam

**Affiliations:** ^1^Department of Ophthalmology and Visual Sciences, The Chinese University of Hong Kong, Hong Kong, Hong Kong SAR, China; ^2^Hong Kong Eye Hospital, Kowloon, Hong Kong SAR, China; ^3^Department of Ophthalmology and Visual Sciences, Lam Kin Chung, Jet King-Shing Ho Glaucoma Treatment and Research Centre, The Chinese University of Hong Kong, Hong Kong, Hong Kong SAR, China; ^4^Department of Ophthalmology and Visual Sciences, The Prince of Wales Hospital, Hong Kong, Hong Kong SAR, China; ^5^Jockey Club School of Public Health and Primary Care, The Chinese University of Hong Kong, Hong Kong, Hong Kong SAR, China; ^6^Department of Paediatrics and Adolescent Medicine, The University of Hong Kong, Pok Fu Lam, Hong Kong SAR, China; ^7^Department of Ophthalmology, Hong Kong Children Hospital, Kowloon, Hong Kong SAR, China; ^8^Joint Shantou International Eye Centre of Shantou University and Chinese University of Hong Kong, Shantou, China; ^9^Hong Kong Hub of Pediatric Excellence, The Chinese University of Hong Kong, Hong Kong, Hong Kong SAR, China

**Keywords:** myopia, cost-effectiveness analysis, cost, refractive surgery, pathological myopia

## Abstract

The rising prevalence of myopia is a major global public health concern. Economic evaluation of myopia interventions is critical for maximizing the benefits of treatment and the healthcare system. This systematic review aimed to evaluate the cost-effectiveness of interventions for treating myopia. Five databases were searched – Embase, Emcare, PubMed, Web of Science, and ProQuest – from inception to July 2022 and a total of 2,099 articles were identified. After careful assessments, 6 studies met the eligibility criteria. The primary outcomes of this systematic review were costs, quality-adjusted life years (QALYs), and incremental cost-effectiveness ratio (ICER). The secondary outcomes included utility values and net monetary benefits (NMB). One study determined the cost-effectiveness of photorefractive screening plus treatment with 0.01% atropine, 2 studies examined cost-effectiveness of corneal refractive surgery, and 3 studies evaluated cost-effectiveness of commonly used therapies for pathologic myopia. Corneal refractive surgeries included laser *in situ* keratomileusis (LASIK), femtosecond laser-assisted *in situ* keratomileusis (FS-LASIK), photorefractive keratectomy (PRK), and small-incision lenticule extraction (SMILE). Interventions for pathologic myopia included ranibizumab, conbercept, and photodynamic therapy (PDT). At an incremental cost of NZ$ 18 (95% CI 15, 20) (US$ 11) per person, photorefractive screening plus 0.01% atropine resulted in an ICER of NZ$ 1,590/QALY (US$ 1,001/QALY) (95% CI NZ$ 1,390, 1,791) for an incremental QALY of 0.0129 (95% CI 0.0127, 0.0131). The cost of refractive surgery in Europe ranged from €3,075 to €3,123 ([US$4,046 to $4,109 - adjusted to 2021 inflation). QALYs associated with these procedures were 23 (FS-LASIK) and 24 (SMILE and PRK) with utility values of 0.8 and ICERs ranging from approximately €14 (US$17)/QALY to €19 (US$23)/QALY. The ICER of LASIK was US$683/diopter gained (inflation-adjusted). The ICER of ranibizumab and PDT were £8,778 (US$12,032)/QALY and US$322,460/QALY respectively, with conbercept yielding a saving of 541,974 RMB (US$80,163)/QALY, respectively. The use of 0.01% atropine and corneal refractive surgery were cost-effective for treating myopia. Treating pathologic myopia with ranibizumab and conbercept were more cost-effective than PDT. Prevention of myopia progression is more cost-effective than treating pathologic myopia.

## 1. Introduction

Myopia is the most common ocular condition worldwide. It affected 2,620 million people (34% of the global population) in the year 2020 and is expected to affect 4,758 million people in the year 2050, or approximately half of the world's population by then (1). This is a serious health concern from a personal and societal perspective because myopia, especially high myopia, is associated with impaired vision and potentially blinding pathologies, including myopic macular degeneration (MMD), glaucoma, and retinal detachment (2). For instance, the odds ratio of developing MMD in high myopia was as high as 845 (3). Although individuals with myopia could achieve 20/20 vision with adequate refractive correction, visual impairment due to myopia reduces an individual's quality of life, limits their vocational choices (4, 5), and increases their risk of falls (6). The high prevalence of myopia also leads to profound consequences in terms of social benefits, risks, and costs (7). A recent estimate suggests that the cost of treating and preventing myopia in China is about US$10 billion annually (8).

In 2015, the global potential productivity loss due to uncorrected myopia and MMD were US$ 244 billion and US$ 6 billion, respectively (9). In Singapore, the annual direct cost of treating myopia was US$ 25 million (10) for teenagers and US$ 755 million for adults (11). These expenditures included costs of performing refractive surgery, purchasing spectacles, contact lenses, contact lens solutions, and treating myopic complications. Myopia can be corrected through optical or surgical means. Optical correction includes the use of spectacles or contact lenses. Surgical correction includes photorefractive keratectomy (PRK), transepithelial photorefractive keratectomy (T-PRK), laser epithelial keratomileusis (LASEK), epipolis laser *in situ* keratomileusis (Epi-LASIK), laser *in situ* keratomileusis (LASIK) with the flap created with either a mechanical microkeratome or femtosecond-based microkeratome (FS-LASIK), femtosecond lenticule extraction (FLEx) and small-incision lenticule extraction (SMILE). Over the years, several interventions for controlling myopia progression have been studied. These include the use of low concentration eye drops, orthokeratology, defocus modifying lenses, bifocal lenses, multifocal lenses, and increased outdoor times. Amongst these interventions, atropine was found to be the most efficacious (12).

An economic assessment of health-care interventions offers useful information for evidence-based advocacy, policy-making, and patient-care decisions (13). Given the burden on health care resources and the high prevalence of myopia, objective economic evaluation of myopia treatment is essential to maximize beneficial outcomes. The economic benefits of interventions for myopia have not yet been systematically examined. Widespread adoption of myopia interventions may be hindered by the lack of evidence on economic evaluations. This systematic review aimed to examine the cost-effectiveness of interventions for myopia and its complications. The present review examines interventions that represent the lifetime spectrum of myopia, including prevention of myopia progression, correction of refractive error, and treatment of pathologic myopia. Correction of myopia and controlling progression in children is crucial to preventing visual impairment from pathologic myopia in adulthood.

## 2. Methods

We conducted this systematic review in accordance with the preferred reporting items for systematic reviews and meta-analyses (PRISMA) guidelines (Appendix Table 1) and is registered with PROSPERO (CRD42022309196).

### 2.1. Eligibility criteria

The inclusion of studies was limited to full original economic evaluations (i.e., cost-effectiveness, cost-utility) of myopia interventions and were published in English. We included all economic evaluations regarding myopia with no restriction to age. We excluded publications that only evaluated costs, were reviews, reports, comments, letters, editorials, abstracts only or did not report the outcome of interest. Costs, quality-adjusted life years (QALYs), and the incremental cost-effectiveness ratio (ICER) were the primary outcomes, while secondary outcomes were utility values and net monetary benefits (NMB). QALY is a summary measure used to assess the effectiveness of an intervention. QALYs are calculated using utility values, which are assessments of health-related quality of life evaluated on a scale where perfect health is valued as 1 and death as 0. The ICER is a summary measure that represents the economic value of an intervention, compared with an alternative. The NMB represents the monetary value of an intervention when a willingness to pay threshold is known (14).

### 2.2. Search methods

We searched five databases, including the Ovid platform (Embase and Emcare), PubMed, Web of Science, and ProQuest, from inception to July 2022. Search keywords included “cost” or “cost-effectiveness” or “economic evaluation” and “myopia” or “nearsightedness” or “shortsightedness”. Additional information about the search method is provided in the Appendix 3. We modified the search terms and conducted an additional search with specific myopia progression interventions such as outdoor activity, orthokeratology lenses, contact lenses, and spectacle lenses (Appendix 3.6).

### 2.3. Study selection and data collection

The titles, abstracts, and full-text articles were reviewed for inclusion using data extraction forms created in Covidence. Data extracted comprised of first author, year of publication, location of study, interventions, start age, model used, time-horizon, perspective, discount rate, and outcomes. Data extracted from the included studies were analyzed using narrative synthesis.

### 2.4. Risk of bias assessment

The methodological quality of the included studies was assessed by the critical appraisal tool developed by Drummond et al. (15) for assessing economic evaluations. This appraisal tool consists of 10 appraisal questions covering the description of interventions, the measure of costs and outcomes, clinical effectiveness, and uncertainty (sensitivity analysis and generalizability). Study questions and objectives were clearly stated in all studies, along with comprehensive description of alternatives for which cost-effectiveness was determined. Evidence used to derive effectiveness estimates had to be clearly reported in all studies, each of which addressed uncertainties by conducting sensitivity analysis to determine the impact of varying study inputs on the results. Methodological quality of each study is summarized in Appendix 1.

## 3. Results

**Figure 1:**
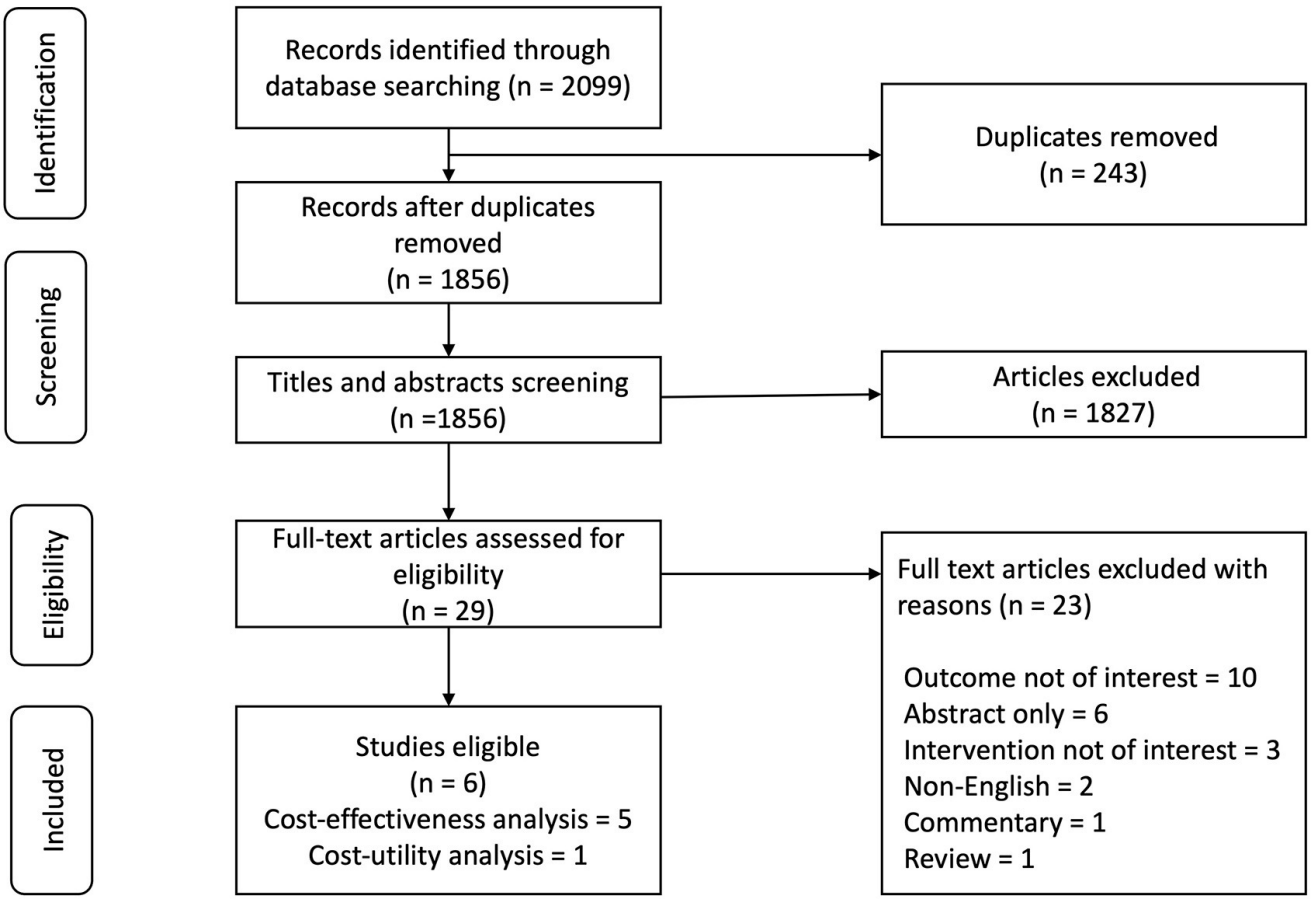
Flow chart of study retrieval and selection.

Figure 1 shows a flow chart of the study retrieval and selection procedure. In total, 2,099 articles were identified from the search strategy. Covidence was used to manage the retrieved studies and for duplicate removal. 1,856 articles remained after the removal of duplicates. After abstracts and titles screening, 1,827 articles failed to meet the inclusion criteria. Finally, a full-text review of 29 articles was conducted, and 23 were excluded because they did not measure relevant outcomes (*n* = 10), had abstracts only (*n* = 6), did not evaluate relevant interventions (*n* = 3), were not in English (*n* = 2), was a commentary (*n* = 1) or a review (*n* = 1). Overall, 6 studies met the criteria for inclusion.

The studies were conducted between the years 2002 and 2022 in Spain, the United Kingdom (UK), the United States of America (USA), China, Germany, and New Zealand (Table 1). All studies used local currencies to report their analyses. In reporting our study results, all currencies were converted into US dollars (USD) at official conversion rates as of 1st August 2022 and older costs were adjusted to 2021 inflation. Additional details of conversion rates can be found in the Appendix 4. Two studies evaluated the cost-effectiveness of refractive surgeries (17, 18), one study evaluated the use of 0.01% atropine to treat children who screened positive for myopia (16), and three studies evaluated the cost-effectiveness of treating pathological myopia (19–21). The studies reported varying time horizons ranging from 1 year to a lifetime. Five studies were cost-effectiveness analysis (CEA) and one study was a cost-utility analysis (CUA) (21). Study perspectives were specified as societal, healthcare, payer, patient, and insurer. One study used the societal perspective (16). The payer perspective was used in four of the studies, except one that used the healthcare perspective (20). One study each took both “healthcare and payer” (17), “healthcare and patient” (18), and “patient and insurer” (21) perspectives. Three studies used Markov model (19, 20), two studies used a decision tree model (17, 21), and one did not specify the model used (18). Outcomes reported in these studies were costs, utility values, QALYs, and ICERs, with one study also reporting the net monetary benefit (NMB) (20). The most common outcome of the cost-effectiveness summary was cost per QALY, except for one study whose outcome was cost per refractive gain unit (18). One study by Javit and Chiang (22) that examined the socioeconomic aspects of laser surgery met the inclusion criteria but was excluded because the outcome of effectiveness used was unclear (22).

**Table 1:** Summary of included studies.

	**Economic assessment**	**Perspective, year of costs**	**Country**	**Interventions**	**Start age (years)**	**Time-horizon (years)**	**Discount rate**	**Model**	**Outcomes**	**Study conclusions**
**Childhood myopia control**										
Hong et al. (16)	CEA	Societal, 2021	New Zealand	Photorefractive screening plus atropine 0.01% vs. corrective lenses	11	Lifetime (80) years	3%	Markov model	Costs, Utility values, QALY, ICER	Photorefractive screening plus atropine 0.01% for 2 years is cost-effective compared to giving corrective lenses only
**Myopia correction**										
Balgos et al. (17)	CEA	Payer and healthcare, 2020	Spain	SMILE, vs. FS-LASIK vs. PRK	30	30	3%	Decision tree model	Costs, Utility values, QALY, ICER	SMILE, FS-LASIK and PRK are cost-effective when performed between the ages of 20 and 60 years
Lamparter et al. (18)	CEA	Healthcare and patient, NS	Germany	LASIK vs. no treatment	NS	NS	NS	NS	Costs, Utility values, QALY, ICER	LASIK is cost-effective for myopia correction
**Pathologic Myopia**										
Cui et al. (19)	CEA	Payer, NS	China	Conbercept vs. ranibizumab	NS	10	3.5%	Markov chain model	Costs, Utility values, QALY, ICER	Conbercept is more cost-effective than ranibizumab for pathologic myopia, from the Chinese payer's perspective
Claxton et al. (20)	CEA	Healthcare, 2011	United Kingdom (UK)	Ranibizumab vs. PDT vs. observation	55	Lifetime	3.5%	Markov model	Costs, Utility values, QALY, ICER, NMB	Ranibizumab is dominant over PDT for pathologic myopia in the UK healthcare setting and cost effective compared with observation only
Sharma and Bakal (21)	CUA	Insurer and patient, NS	USA	PDT	50	1	3%	Decision analysis model	Costs, Utility values, QALY, ICER	PDT was not cost-effective when time horizon was 1 year but cost-effective when time-horizon increased to 10 years

CEA, cost-effectiveness analysis; CUA, cost-utility analysis; NS, not specified; LASIK, Laser *in situ* keratomileusis; FS-LASIK, femtosecond laser-assisted *in situ* keratomileusis; PRK, photorefractive keratectomy; PDT, photodynamic therapy; QALY, quality-adjusted life years; ICER, incremental cost-effectiveness ratio.

### 3.1. Childhood myopia control and preventing myopia progression

Prevention of myopia progression is essential in children because it leads to visual impairment later in life, especially for children at high risk. Several myopia progression interventions have been studied over the years, including the use of pharmacological agents, special contact lenses, and spectacle lenses (12). To date, only one study evaluated the cost-effectiveness of childhood myopia control (16).

Hong et al. (16) determined the cost-effectiveness of a hypothetical photorefractive myopic screening program plus offering low-dose atropine (0.01%) in the New Zealand (NZ) setting (16). Based on a lifetime horizon (80 years) at a 3% discount rate and a Markov model simulation, the impact of screening plus the use of atropine compared to usual care (corrective lenses) in 11-year-old children was assessed. Costs included costs of consultation, optometry visits, corrective lenses, screening, monitoring, drugs, and low vision costs. The cost of myopia was directly related to its severity. For instance, the cost of myopia increased from NZ$ 264 (US$ 166) to NZ$ 1,923 (US$ 1,210) in pathologic myopia per year. A further progression to blindness resulted in an estimated cost of NZ$ 3,846 (US$ 2,420) per year. Utility values were specified as disability weights. A disability weight is a weight factor that reflects the relative severity of a health state, quantified on a scale from 0 (perfect health) to 1 (death). Myopia was associated with a disability weight of 0.003. At an incremental cost of NZ$ 18 (95% CI 15, 20) (US$ 11) per person, photorefractive screening plus 0.01% atropine resulted in an ICER of NZ$ 1,590/QALY (US$ 1,001/QALY) (95% CI NZ$ 1,390, 1,791) for an incremental QALY of 0.0129 (95% CI 0.0127, 0.0131). At a willingness-to-pay (WTP) threshold of NZ$ 58,000 (US$ 36,497), 0.01% atropine was cost-effective in New Zealand. According to this study, 816 cases of high myopia, 462 cases of pathological myopia, and 7 cases of blindness (for every 100,000 screened) could be prevented with the use of 0.01% atropine if all patients, who were at risk of myopia progression, accepted the treatment. To prevent 1 case of blindness, 14,286 children needed to be screened. In sensitivity analysis, it was more cost-effective to screen and treat children at an earlier age, i.e., at 5 years old rather than at 11 years old. Additionally, the intervention became more cost-effective when life expectancy increased from 80 to 95 years.

### 3.2. Myopia correction

Corneal refractive (keratorefractive) surgeries correct myopia by reshaping the cornea to reduce its refractive power and are alternatives to spectacles or contact lenses for optical correction of refractive errors (23). In general, these procedures can be classified into three types: corneal surface ablation surgery, corneal stromal ablation surgery, and refractive corneal lenticule extraction. Several surface ablation procedures available include photorefractive keratectomy (PRK), transepithelial photorefractive keratectomy (T-PRK), laser epithelial keratomileusis (LASEK), and epipolis laser *in situ* keratomileusis (Epi-LASIK) (24). Corneal stromal ablation surgeries (including laser *in situ* keratomileusis [LASIK] and femtosecond laser-assisted *in situ* keratomileusis [FS-LASIK]) involve the creation of corneal flap (25). Whereas, refractive corneal lenticule extraction procedures [including femtosecond lenticule extraction (FLEx) and small-incision lenticule extraction (SMILE)] do not require flap creation (26).

Two studies evaluated the cost-effectiveness of refractive surgeries. Balgos et al. (17) compared the cost-effectiveness of three corneal refractive procedures (PRK, FS-LASIK, and SMILE) for treating myopia and myopic astigmatism (17). With an annual discount rate of 3%, a decision tree model was used to project costs and outcomes associated with these procedures over a period of 30 years from the perspective of the payer (patient) and healthcare system (the eye center). From the payer's perspective, only costs directly incurred by patients were included. On the contrary, from the healthcare perspective, both direct and indirect costs incurred by the eye center were included. Direct costs included costs of consultation, screening for refractive surgery, postoperative medications, managing complications, medical equipment, and personnel. Costs associated with the procedures were high, with FS-LASIK being the most expensive. The annual costs of SMILE, PRK, and FS-LASIK were estimated at € 9,979 (US$ 10,212), € 6,868 (US$ 7,028), and € 10,314 (US$ 10,555), respectively. Over a period of 30 years, these costs were expected to increase to € 25,854 (US$ 26,456), € 22,444 (US$ 22,967), and € 25,889 (US$ 26,493), respectively. The annual cost of maintaining the operating facilities for corneal refractive surgery was € 403,000 (US$ 412,390) for SMILE, € 353,000 (US$ 361,225) for PRK, and € 403,000 (US$ 412,390) for FS-LASIK. Corneal refractive procedures improved utility values. The utility is a measure of patient-perceived quality of life associated with a health state, quantified on a scale from 0.00 (death) to 1.00 (perfect health). With a baseline average utility of 0.61 for myopic patients before undergoing refractive surgery, the weighted average utility values improved to 0.80 for patients who underwent SMILE or PRK and 0.77 for patients who underwent FS-LASIK. These utilities produced corresponded to QALYs of 24 for SMILE and PRK, and 23.1 for FS-LASIK. Hence, the ICER for SMILE, PRK, and FS-LASIK were approximately € 14 (US$ 14)/QALY, € 18 (US$ 18)/QALY, and € 15 (US$ 15)/QALY, respectively. In sensitivity analysis, the ICERs ranged from € 8 to € 19 (US$ 8–19)/QALY for SMILE, € 11 to € 31 (US$ 11 to 31)/QALY for PRK, and € 9 to € 25 (US $9 to 26)/QALY for FS-LASIK. These estimates were below the WTP thresholds specified, and the study concluded that these corneal refractive surgeries are cost-effective.

The cost-effectiveness of LASIK compared with no treatment in moderate myopia was examined by Lamparter et al. (18) from a health care service provider perspective (18). Accordingly, only direct costs were included. The discount rate, time horizon, and WTP threshold in this study were not reported. The study determined cost-effectiveness with a model that was not specified. Costs included the direct cost of LASIK treatment and treatment of surgical complications. The direct cost of primary LASIK procedure was estimated at € 2,426 (US$ 3,192) per eye. Complications associated with LASIK resulted in an additional cost of € 648 (US$ 853), increasing the total direct cost to € 3,075 (US$ 4,046). The outcome of effectiveness was refractive gain due to conventional LASIK procedures. With the aid of a meta-analysis, LASIK was reported to produce a clinical benefit of 5.93 dioptres (D) and an ICER of € 519 (US$ 683/gained refractive benefit unit. A deterministic sensitivity analysis varying costs by ± 10% and meta effects of refractive gain within 95% confidence intervals resulted in an ICER ranging from € 445 (US$ 585) per gained diopter to € 600 (US$ 789) per gained dioptre. The study concluded that LASIK was a cost-effective procedure for myopia treatment.

### 3.3. Treatment of myopia complications

Myopia progression can result in pathologic myopia that is characterized by extreme, continuous axial elongation and leads to degenerative alterations in the posterior segment of the eye (27). Pathologic myopia is one of the most common causes of blindness worldwide, affecting up to 3% of the world's population (28). Three studies evaluated the cost-effectiveness of treating pathologic myopia with intravitreal injection of anti-vascular endothelial growth factor (anti-VEGF) (e.g., ranibizumab, conbercept), and photodynamic therapy (PDT) (Tables 1, 2). In all three studies, pathologic myopia referred to choroidal neovascularization secondary to high myopia. The cost-effectiveness of interventions for myopia-related macular degeneration, retinal detachment, cataracts, and glaucoma were not studied despite their association with myopia (3).

**Table 2:** Summary of outcome values from included studies.

**Study**	**Time-horizon**	**Interventions**	**Costs**	**Incremental costs**	Effectiveness^*^	**Incremental effectiveness**	**Incremental cost-effectiveness ratios (ICERs)**	**Utilities**	**Net monetary benefits (NMB)**
**Childhood myopia control**									
Hong et al. (16)	Lifetime (80) years	Photorefractive screening plus atropine 0.01% vs. corrective lenses	NA	NZ$ 18	NA	0.0129	1,590/QALY	NA	NA
**Myopia correction**									
Balgos et al. (17)	30 years	SMILE	€ 25,854	NA	24	NA	14/QALY	0.8	NA
		FS-LASIK	€ 25,889	NA	23.1	NA	15/QALY	0.77	NA
		PRK	€ 22,444	NA	24	NA	18/QALY	0.8	NA
Lamparter et al. (18)	NS	LASIK	€3,075	-	5.930D	-	519/dioptre gained	NA	NA
**Pathologic myopia**									
Cui et al. (19)	10 years	Conbercept	RMB 222,648	RMB −15,411	7.528	0.029	−541,974/QALY	^+^	NA
		Ranibizumab	RMB 238,059	-	7.499	NA	NA	^+^	NA
Claxton et al. (20)	Lifetime	Ranibizumab	£12,866	NA	12.99	NA	NA	^+^	NA
		PDT	£14,421	NA	12.6	NA	NA	^+^	NA
		Observation	£8,163	NA	12.45	NA	NA	^+^	NA
		Ranibizumab vs. PDT	-	£-1,555	-	0.39	Dominant	NA	£ 9,289
		Ranibizumab vs. observation	-	£4,703	-	0.54	8,778/QALY	NA	£ 6,013
Sharma and Bakal (21)	1 year	PDT (4 treatments)	-	$9,120	-	0.037	246,486/QALY	^+^	NA

NS, not specified; NA, not applicable; D, dioptre.

* Effectiveness is measured in quality-adjusted life years (QALYs), unless specified.

+ Utility values reported were based on best corrected visual acuity.

Sharma and Bakal (21) investigated the cost-effectiveness of PDT for treating pathologic myopia from the patient and insurer perspectives. This cost-utility analysis was based on the case of a 50-year-old monocular patient with pathologic myopia who received PDT for subfoveal choroidal neovascular membrane over a year. At an annual discount rate of 3%, the incremental cost of PDT was estimated at US$ 1,998 (inflation-adjusted = US$ 3009), considering the cost of physician reimbursement, the cost of fluorescein angiography, and the cost of dye. Utility values based on visual acuity were used to determine QALYs. PDT yielded a QALY of 0.037 when compared to no treatment. ICER of PDT increased with an increasing number of treatments required (i.e., as the number of treatments required increased, PDT became less cost-effective). For instance, an ICER of $ 54,000 ($ 81,336)/QALY was obtained when only one treatment was required. This ICER increased to $ 214,085 ($ 322,460)/QALY for an average of 3.4 treatments of PDT and $ 246,486 ($ 371,263)/QALY when a patient required 4 treatments over the same period. These values exceeded the WTP threshold, indicating that PDT was not cost-effective. However, in sensitivity analysis, PDT became cost-effective when the time horizon was extended to 10 years, yielding an ICER of $ 20,000 ($ 30,124)/QALY.

The cost-effectiveness of ranibizumab and PDT compared with observation alone for treating myopic choroidal neovascularization (CNV) was assessed by Claxton et al. (20). Adapting a UK healthcare perspective, analysis was performed with a Markov model over a lifetime time horizon at an annual discount rate of 3.5%. Costs included costs of treatment, monitoring, management of adverse events, ophthalmologist consultations, cost of optical coherence tomography (OCT), injecting ranibizumab or performing PDT by ophthalmologist, and long-term cost of blindness. The lifetime costs of managing myopic CNV by ranibizumab [£ 12,866 (US$ 17,636)] was slightly lower than PDT [£ 14,421(US$ 19,767)] but higher than observation only [£ 8,163 (US$ 11,189)]. Health utility values were determined based on whether patients were treated in their better or worse seeing eyes. In the absence of visual impairment, this value was the same (0.85), irrespective of which eye received treatment. Utility values associated with treating the worse seeing eye were higher than treating the better seeing eye. Patients who read <25 letters had a utility of 0.353 and 0.750 when treated in their better and worse seeing eyes, respectively. Ranibizumab gained more QALYs (12.99) than PDT (12.60) and observation alone (12.45), resulting in an ICER of £ 8,778 (US$ 12,032)/QALY. Only this study reported the net monetary benefit (NMB). Ranibizumab gained a NMB of £ 9,289 (US$ 12,733) at a WTP threshold of £ 20,000 (US$ 27,414)/QALY. From a UK healthcare perspective, ranibizumab dominated PDT when compared with observation only. Hence, treating myopic CNV with ranibizumab was more cost-effective.

Cui et al. (19) adapted a real-world scenario and a randomized controlled trial (RCT) scenario to examine the cost-effectiveness of conbercept and ranibizumab for treating pathologic myopia from a payer's perspective in China. A Markov model was used for this study over a time horizon of 10 years, with a discount rate of 3.5% per year. Only direct medical costs of drugs, inspection, surgery, nursing, and treatment were included. Single conbercept and ranibizumab injections were estimated at 5,550 RMB (US$ 821) and 5,700 RMB (US$ 843), respectively. The number of injections in a year was approximately 2 times in the real-world scenario and 4 times in the RCT scenario. Over a 10-year period, the total cost of ranibizumab was 117,198 RMB (US$ 17,335) and conbercept was 106,587 RMB (US$ 15,765) in the real-world scenario. Whereas, in the RCT scenario over a 10-year period, the total cost of ranibizumab and conbercept were 238,059 RMB (US$ 35,211) and 222,648 RMB (US$ 32,932), respectively. QALYs were determined by utility values associated with best corrected visual acuities (BCVA) at different health states of pathologic myopia. Health utility values decreased from 0.7562 for patients with no visual impairment to 0.3254 for patients with blindness. Ranibizumab and conbercept gained 7.499 and 7.528 QALYs, respectively, in both a real world and an RCT scenario. Conbercept was found to be more cost-effective than ranibizumab for treating pathologic myopia in China. Compared with ranibizumab, the ICER of conbercept was−373,185 RMB (US-55,198)/QALY and−541,974 RMB (US-80,162)/QALY in real life and an RCT scenario, respectively.

## 4. Determinants for cost-effectiveness

All studies included in this systematic review conducted deterministic sensitivity analyses, which comprised of one and/or two-way sensitivity analyses. Two studies conducted only deterministic analysis (17, 18) and 4 conducted both deterministic and probabilistic sensitivity analyses (19–21).

Different scenarios that had an impact on cost-effectiveness were cost, utility gain, time-horizon, efficacy of 0.01% atropine, and the number of treatments required. 0.01% atropine became less cost-effective when its efficacy was reduced, and more cost-effective with extended time-horizon (16). Refractive surgery became more cost-effective over a longer period (i.e., when surgery was performed earlier) (17). Concerning treatments of myopic CNV (20), cost-effectiveness was greatly influenced by utility gain for the worse seeing eye, number of anti-VEGF injections, and follow-up visits. For maximum utility gain in the worse seeing eye, ranibizumab became more cost-effective than the base-case estimate compared to PDT or observation only. An increase in the number of treatments in year 2 had a more substantial impact on cost-effectiveness of ranibizumab when compared to PDT. The number of ranibizumab treatments given in year 1 was approximately 3.5 compared to 3.4 for PDT. Ranibizumab remained cost-effective when the number of treatments was assumed to be 12 compared with an average of 3.4 treatments of PDT but ceased to be cost-effective when 11 or more injections were given in year 2. Sharma and Bakal (21) demonstrated that PDT was not cost-effective regardless of the number of treatments required over a time horizon of 1 year but became cost-effective when the time horizon was increased to 10 years.

## 5. Discussion

In this systematic review, we analyzed the cost-effectiveness of various interventions for myopia, including prevention of myopia progression, refractive correction of myopia, and treatment of myopia complications (i.e., pathologic myopia in highly myopic patients). Myopia progression is associated with potentially blinding complications related to high myopia (2). Various interventions to control myopia progression have been studied over the years. These include the use of pharmacological agents (atropine, pirenzepine, timolol, and cyclopentolate), contact lenses (orthokeratology, soft contact lenses, rigid gas-permeable contact lenses, and peripheral defocus modifying contact lenses), spectacle lenses (single vision spectacle lenses, progressive addition spectacle lenses, prismatic bifocal spectacle lenses, peripheral defocus modifying spectacle lenses), and lifestyle modification (e.g., spending more time outdoors) (29–33). Among these options, atropine eye drops was shown to be the most efficacious treatment modality (12), and 0.05% atropine was suggested to be the optimal concentration (34).

Despite the availability of effective interventions to retard myopia progression in children, the cost-effectiveness is unknown. Pathologic myopia and blindness are associated with a high cost. For instance, Germany spent an estimated € 49.6 billion annually on blindness and moderate-to-severe visual impairment (35). The annual direct treatment cost of patients with myopic CNV was about four times higher than that of high myopia subjects without CNV (36). Identifying and treating myopic children should theoretically reduce disease severity and the risk of blinding complications. Hence, reduce the cost of treating myopic-related conditions in their adulthood. For example, screening 100,000 children and providing treatment to retard myopic progression could avoid 816 cases of high myopia, 462 cases of pathologic myopia, and 7 cases of blindness (16). Pathologic myopia incurred huge additional treatment costs; the cost increment was over 100% if myopia progressed to the pathologic state (US$ 166 to US$ 1,210) and a further 100% increment when pathologic myopia progressed to blindness (US$ 1,210 to US$ 2,420). Understanding the cost-effectiveness of preventing myopic progression and identifying a cost-effective intervention is crucial for health care policy-making and patients' quality of life.

We only identified one study [Hong et al. (16)] that demonstrated the cost-effectiveness of retarding myopia progression; by treating 11-year-old children with 0.01% atropine if they were screened positive for myopia. The approach was sensitive to the age of initiating treatment, life expectancy, and the efficacy of 0.01% atropine in reducing progression to high myopia. The intervention would be more cost-effective if treatment were started earlier, with more effective treatment, and a life expectancy increase from 80 to 95 years old. Furthermore, maximal myopia progression occurs between the age of 6 to 10 years (37); their approach of screening myopic children at the age of 11 years for intervention may not identify the highest risk group. Given the lack of CEA in the field, more CEA with different health care settings is warranted. For instance, our recent studies demonstrated that 0.05% atropine was more efficacious than 0.01% atropine (38, 39); the cost-effectiveness of the two treatments has not been compared. According to the sensitivity analysis of Hong et al. (16), we expect myopia screening and prompt initiation of a more efficacious concentration of atropine at an earlier age in high prevalence regions (e.g., using 0.05% atropine at 4 to 5 years old in East Asian countries) (29, 38, 39) will be an even more cost-effective approach. This is especially true for Asian countries known for their high and rising prevalence of myopia in children, which vaticinates the growing burden of myopia-related problems in their health care systems. For instance, the reported prevalence of myopia in Hong Kong was 25% among 6 to 8 years old children and 72.2% among adults (40). Standing as a region with one of the longest life expectancies worldwide, such a CEA based on level I evidence data will be pivotal for health care policy and formulation of treatment guidelines.

Corneal refractive surgeries (PRK, LASIK, FS-LASIK, and SMILE) were cost-effective based on the models included in this systematic review. This is consistent with the economic evaluations that showed PRK was more cost-effective than corrective lenses if the surgery was performed at an earlier age (17) and the cost of surgery and treatment failure were reduced (22). Although these procedures are cost-saving (41) with a low rate of complications (17, 42), they could lead to irreversible damage and much higher treatment costs if complications occur. The complications include infections, inflammation, light sensitivity, central islands, over or under correction, haze, dry eyes, and retinal detachment. The cost of LASIK could increase by € 648 (US$ 853) or 27% from € 2,426 (US$ US$ 3,192) with uneventful surgery to € 3,075 (US$ 4,046) if any complication occurs (18, 43). Refractive surgery is an elective procedure (44) (given the high cost and potential sight-threatening complications) and myopia is usually corrected by spectacles or contact lenses. The average cost of spectacles, soft contact lenses, and rigid lenses were € 204 (US$ 286), € 184 (US$ 258), and € 160 (US$ 224), respectively (45), compared with € 2,426 (US$ 3,192) of LASIK (18, 43). Furthermore, spectacle or contact lenses may require cleaning, replacement, or repair, which could incur additional fees. It was estimated that over a period of 30 years, the costs (direct and indirect) of LASIK, eyeglasses, and contact lenses would be € 3,792 (US$ 5,319), € 2,197 (US$ 3,082), and € 11,697(US$ 16,409), respectively (45). The drawback of correcting myopia with spectacle, contact lenses, or refractive surgery is that they do not retard myopia progression and the related complications.

Three studies (19–21) analyzed the cost-effectiveness of treating pathologic myopia with intravitreal injection of anti-VEGF (ranibizumab and conbercept) and PDT in different countries. In the UK, ranibizumab was more cost-effective than PDT when compared to observation alone (20). Ranibizumab is dominant over PDT for treating pathologic myopia because the former was more successful in visual improvement (46) and was less expensive [£ 12,866 (US$ 17,636) for ranibizumab vs. £ 14,421 (US$ 19,767) for PDT] (20). In China, ranibizumab was less cost-effective than conbercept for treating pathologic myopia from a payer's perspective. Conbercept showed significant cost-effectiveness even when the costs and the number of injections varied. Ranibizumab was about 49.6% likely to be cost-effective in China, according to the sensitivity analysis (19). The variation of economic settings and clinical practice is known to influence the results of CEA. The differences in cost-effectiveness of ranibizumab in the UK and China may be due to the differences in the economic settings of these countries. Ranibizumab, for example, costs more in the UK (US$ 17,636) than in China (US$ 17,335) (19, 20). No other study assessed the cost-effectiveness of conbercept in regions other than China because it was only approved for use in China at the time of this study. The three studies (19–21) differed in their settings and the type of comparators used, making it difficult to compare their cost-effectiveness. Nevertheless, ranibizumab and conbercept seem to be better options than PDT (19, 20), given that PDT was less cost-effective than observation alone (16, 18) and may lead to long term chorioretinal atrophy and visual loss in some patients (47, 48). Another potential anti-VEGF that has been shown to be safe and efficacious for treating pathologic myopia is aflibercept (49). However, aflibercept is reported to be the most expensive among the clinically available anti-VEGF drugs. Conbercept on the other hand is safe, efficacious, and cost-effective for treating pathologic myopia in China. No study that compared the cost-effectiveness of aflibercept with conbercept for treating pathologic myopia was identified. Further study is necessary to compare their cost-effectiveness.

Our review is the first systematic review that summarizes the cost-effectiveness of treating myopia. These studies were region-specific and not generalizable on their own. Therefore, we intended to draw them together and provide a broader view of the cost-effectiveness of treating various aspects of myopia. Notably, even though treating pathologic myopia is more expensive, preventing myopia progression and the associated complications also requires screening and treating a large number of children with atropine for an extended period of time. Having the heterogeneous studies presented side-by-side, we showed that preventing myopia progression at an early age is likely to be more cost-effective than treating pathologic myopia in adulthood. The use of 0.01% atropine for myopia progression produced an ICER of $ 1001/QALY versus between $ 12,852/QALY to $ 246,486/QALY for treating pathologic myopia (16, 20, 21). More costs are incurred as myopia progresses to pathological states and even blindness. Using 0.01% atropine to prevent myopia progression may reduce the undesirable eventualities associated with substantial additional costs (16). Furthermore, despite the lower cost-effectiveness estimation related to refractive surgery ($ 14/QALY to $ 18/QALY), 0.01% atropine appeared to be superior to refractive surgery with the lower treatment costs and the additional benefit of preventing myopia complication and blindness. Nonetheless, formal evaluation is necessary to confirm the cost-effectiveness of early myopia prevention

The systemic review has some limitations. First, there were a limited number of studies, with two (15, 16) reporting data from approximately 20 years ago. The lack of studies included in our review according to our selection criteria, coupled with the changing value of money and the health care environment, highlight the need for more CEA to evaluate myopia control. Second, the results of the studies in this review were heterogeneous. However, the interventions and the targeted patients' group represent the lifetime spectrum of the “myopia continuum”; drawing a relationship between these results provides insight into the future research direction and management approach in the field of myopia. Third, the data presented in our study were region-specific and not generalizable. These studies were conducted in different regions with varying economic environments and had different comparators, making it difficult to compare their results. In addition, since most studies were conducted in developed countries, the applicability of the result in low-income countries requires further investigation. Lastly, although corneal refractive surgeries are cost-effective, the most cost-effective approach still remains unknown. The only study that compared SMILE, PRK, and FS-LASIK could not perform a statistical analysis between the cost-effectiveness values, hence no conclusion on the better surgical approach could be made. Although LASIK is cost-effective, it remains unclear, however, the WTP threshold and the time period over which the analysis was conducted.

## 6. Conclusion

In conclusion, low concentration atropine (0.01%) and corneal refractive surgery are cost-effective options for treating myopia. Ranibizumab and conbercept are cost-effective for treating pathologic myopia. 0.05% atropine can effectively slow or halt myopia progression in children with acceptable side effects and potentially reduce the cost of treating myopia complications in adulthood. Currently, there is a limited number of economic evaluations for the treatment of myopia; the cost-effectiveness of early interventions to prevent myopia progression in children is unknown. With the rising prevalence of children with myopia, a comprehensive cost-effectiveness analysis for the topic is necessary.

## Author contributions

SA: conception of the study, study screening, drafting of the manuscript, and final approval. PC: conception of the study, study screening, co-supervised the work, drafting of the manuscript, and final approval. YZ, ZH, BY, PI, CT, and LC: critical review of manuscript and final approval. XZ: critical review of manuscript, co-supervised the work, and final approval. CP: conception of the study, critical review of manuscript, drafting of the manuscript, and final approval. JY: conception of the study, critical review of manuscript, supervised the work, and final approval. All authors contributed to the article and approved the submitted version.
